# Thin-Film Quantum Dot Photodiode for Monolithic Infrared Image Sensors †

**DOI:** 10.3390/s17122867

**Published:** 2017-12-10

**Authors:** Pawel E. Malinowski, Epimitheas Georgitzikis, Jorick Maes, Ioanna Vamvaka, Fortunato Frazzica, Jan Van Olmen, Piet De Moor, Paul Heremans, Zeger Hens, David Cheyns

**Affiliations:** 1 IMEC, Kapeldreef 75, B-3001 Leuven, Belgium; Epimitheas.Georgitzikis@imec.be (E.G.); Ioanna.Vamvaka.ext@imec.be (I.V.); Fortunato.Frazzica@imec.be (F.F.); Jan.VanOlmen@imec.be (J.V.O.); Piet.DeMoor@imec.be (P.D.M.); Paul.Heremans@imec.be (P.H.); David.Cheyns@imec.be (D.C.); 2 KU Leuven, Kasteelpark Arenberg 10, B-3001 Leuven, Belgium; 3 Physics and Chemistry of Nanostructures, Ghent University, Krijgslaan 281-S3, B-9000 Ghent, Belgium; Jorick.Maes@UGent.be (J.M.); Zeger.Hens@UGent.be (Z.H.); 4 Center for Nano- and Biophotonics (NB-Photonics), Ghent University, B-9000 Ghent, Belgium; 5 Vrije Universiteit Brussel (VUB–ETRO), Pleinlaan 2, B-1050 Brussel, Belgium

**Keywords:** infrared, imaging, image sensor, quantum dot, PbS, monolithic integration

## Abstract

Imaging in the infrared wavelength range has been fundamental in scientific, military and surveillance applications. Currently, it is a crucial enabler of new industries such as autonomous mobility (for obstacle detection), augmented reality (for eye tracking) and biometrics. Ubiquitous deployment of infrared cameras (on a scale similar to visible cameras) is however prevented by high manufacturing cost and low resolution related to the need of using image sensors based on flip-chip hybridization. One way to enable monolithic integration is by replacing expensive, small-scale III–V-based detector chips with narrow bandgap thin-films compatible with 8- and 12-inch full-wafer processing. This work describes a CMOS-compatible pixel stack based on lead sulfide quantum dots (PbS QD) with tunable absorption peak. Photodiode with a 150-nm thick absorber in an inverted architecture shows dark current of 10^−6^ A/cm^2^ at −2 V reverse bias and EQE above 20% at 1440 nm wavelength. Optical modeling for top illumination architecture can improve the contact transparency to 70%. Additional cooling (193 K) can improve the sensitivity to 60 dB. This stack can be integrated on a CMOS ROIC, enabling order-of-magnitude cost reduction for infrared sensors.

## 1. Introduction

**Figure 1:**
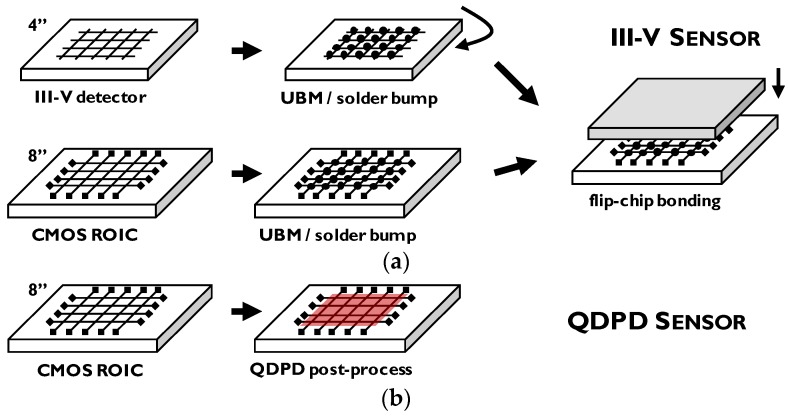
Integration route for a hybrid III–V infrared image sensor (**a**) and a monolithic quantum dot photodiode (QDPD) infrared image sensor (**b**).

Near infrared (NIR) wavelength range (0.7–1.4 μm) provides vital information in fields such as low-light/night vision, surveillance, sorting or biometrics, with content interpretation very similar to visible photography and imaging possible with no additional light source. Unfortunately, the sharply decreasing absorption of silicon around the wavelength of 900 nm (for standard photodiode thickness) prevents further extension of usable quantum efficiency range. At the same time, dedicated infrared sensors are not yet easily accessible due to their significantly higher cost than image sensors based on complementary metal-oxide-semiconductor (CMOS) technology, operating in the visible range. Typically, III–V semiconductor layers are used because of their sufficiently low energy bandgap [1,2]. Since they need to be grown by high-temperature epitaxy (and that on wafer sizes only up to 3–4 inch), already the starting material has orders-of-magnitude higher cost than standard silicon wafers. Moreover, flip-chip hybridization (usually die-to-die) is required which further increases the cost [3]. The ideal, simple solution would be to monolithically integrate an absorber layer directly on top of the silicon-based readout integrated circuit (ROIC, Figure 1). 

**Figure 2:**
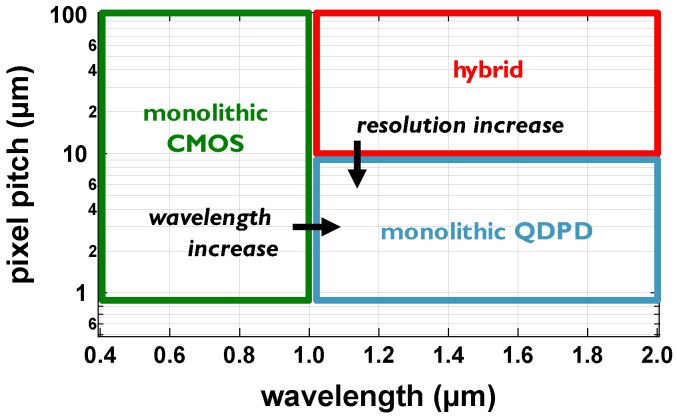
Positioning of a QDPD-based image sensor: higher wavelength than monolithic Si and higher resolution than hybrid alternatives.

Another aspect is the achievable resolution and pixel pitch of infrared image sensors. The traditional hybrid systems are typically limited to small arrays (1 megapixel range) due to small detector wafer size and low throughput. Pixel pitch does not go below 10 μm [4] which is limited by the hybridization process—the solder bumps need sufficient volume for reliable bonding which in turn is limited by the achievable aspect ratio and pixel spacing. With a thin-film active layer integrated monolithically directly on top of the readout circuit, submicron pixel sizes (0.9 μm state-of-the-art for CMOS image sensors [5]) can be achieved (Figure 2). 

Proof-of-concept image sensors with thin-film photodetector active layer have been demonstrated with different materials. Organic absorber integration has already been shown since more than 10 years ago on glass [6,7], flexible plastic foil [8,9] and silicon readout [10]. There have been several demonstrations of organic thin-film photodetector integration on thin-film transistor (TFT) readout circuits, mostly for large area image sensors for X-ray radiography applications [11]. Recently, also perovskite absorbers were successfully integrated [12]. Organic films integration on CMOS ROIC followed a few improvement rounds by the same group, with the latest generation implemented with 0.9 μm pixel size showing the potential of resolution scaling [5,13,14]. Near infrared imaging with solution processed thin-film photodetectors was shown with polymers [15] and quantum dots (QD) [16]. In this work, we describe building blocks for realization of a monolithic image sensor targeted for the infrared range up to the wavelengths close to 2 μm. Quantum Dot Photodetector (QDPD) is the device architecture of choice, with narrow-band PbS colloidal quantum dots [17,18,19] forming the absorber layer. These sensors can be fabricated without flip-chip hybridization of a III–V chip [20]. 

## 2. Materials and Methods 

**Figure 3:**
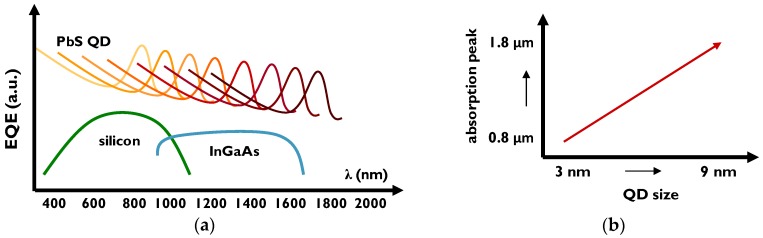
Schematic external quantum efficiency curves for silicon, InGaAs and PbS QD photodetectors (**a**) and indicative absorption peak dependence on quantum dot size (**b**).

PbS quantum dots enable uncooled NIR detection up to 2 μm wavelength, with the absorption peak tunable depending on the nanocrystal size (Figure 3). In this work, we describe two types of quantum dots: Larger (5.5 nm diameter), with the absorption peak at the wavelength of 1440 nm and smaller (3.4 nm diameter), with the peak at 980 nm. The peak can be adjusted according to device specifications, for example with smaller dots to add near infrared bands to hyperspectral visible image sensors and with larger dots to address the spectrum of InGaAs image sensors. 

**Figure 4:**
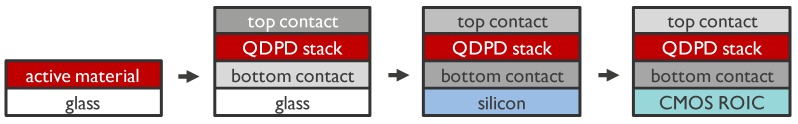
Methodology used for the development of CMOS-compatible QDPD pixel stacks.

In our investigation, we take a stepwise approach to develop a photodetector stack that can be used on top of a readout integrated circuit (ROIC) based on complementary metal-oxide-semiconductor (CMOS) technology (Figure 4). As the first step, we use glass substrates to test the coating feasibility of the colloidal quantum dot solution. Such structure enables optimization of film parameters such as thickness, morphology and uniformity, as well as elaboration of the absorption profile. As the next step, glass substrates with pre-patterned indium tin oxide (ITO) contacts are used to fabricate a photodetector stack with all additional layers, such as electron transport layer (ETL), hole transport layer (HTL) and injection/blocking layers. This stack can be characterized electrically and under bottom illumination (through the substrate). Once the photodiode performance is established, the stack is transferred to Si/SiO_2_ substrates with metal bottom contact to imitate the CMOS ROIC architecture. TiN is used as the contact as it is one of the standard materials in the CMOS flow [21]. Here, we adjust the stack to operate in top illumination condition, which includes changing the top contact to a stack that is as transparent as possible in the wavelength range of interest and tuning the thicknesses of all layers to harness the maximum amount of incoming light [22]. 

The fabrication process starts by cleaning the substrates with a standard detergent—water—solvent procedure. A metal-oxide electron transport layer (ETL) is then deposited to form an inverted architecture of the photodiode (with photo-generated electrons collected at the readout chip side). Examples of this layer are TiO_*x*_ or ZnO (*n*-type semiconductors) that improve electron transport and injection. The quantum dots are deposited by spin-coating from a colloidal solution as a multilayer stack to form a 150 nm thick active layer. The total thickness can be adjusted by the number of sublayers and by the thickness of each sublayer. On top of the stack, an organic (*p*-type polymer) hole transport layer (HTL) is deposited to improve hole transport and injection, followed by a top contact (either thick, opaque metal for bottom illumination or semi-transparent metal for top illumination). 

## 3. Results

### 3.1. Quantum Dot Film

**Figure 5:**
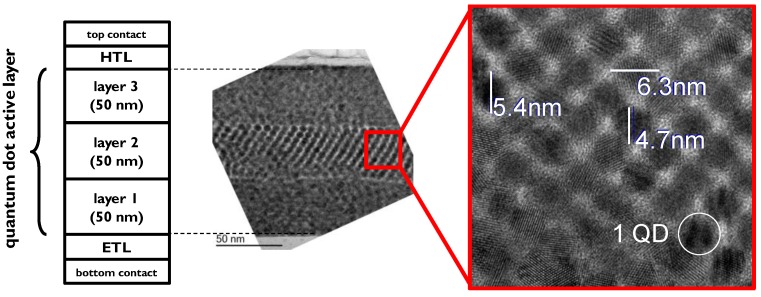
Cross-section (**left**: schematic; **right**: Transmission Electron Micrograph) of a 3-layer active stack based on 5.5 nm PbS quantum dots.

Test substrates with a spin-coated three-sublayer stack of the quantum dot film are characterized with Transmission Electron Microscope (TEM). The inspection reveals perfect crystallinity within each nanocrystal, high level of crystallinity within a single sublayer and random orientation between sublayers (Figure 5). In the high magnification image, one can also see a uniform size distribution of the QDs. 

### 3.2. QDPD on Glass

**Figure 6:**
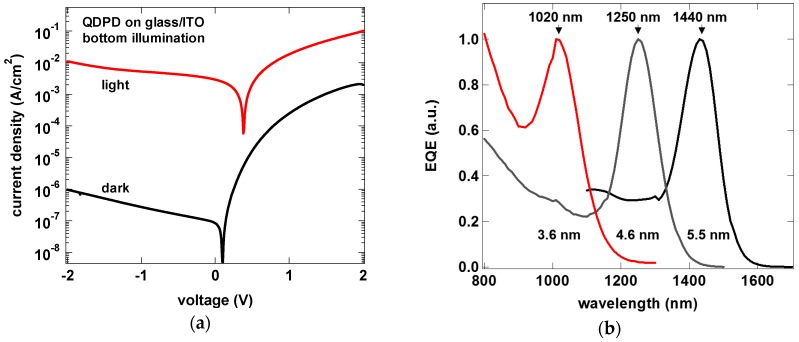
Current density vs. voltage of a bottom illuminated QDPD (**a**) and external quantum efficiency curves for different QD sizes used in the photoactive layer (**b**).

Full photodetector stacks including contacts and transport layers are fabricated on glass/ITO test vehicles. Each 3 × 3 cm^2^ glass substrate has 12 single pixel devices, each with an active area of 0.125 cm^2^. Current density—voltage characterization (Figure 6a) of a baseline stack using 5.5 nm QDs shows dark current density of 1 μA/cm^2^ at −2 V reverse bias voltage. The external quantum efficiency (EQE, Figure 6b) shows that by choosing the size of the quantum dot in the active layer, the absorption peak can be tuned in a wide wavelength range (here, 1020 nm, 1250 nm and 1440 nm). The full width at half maximum (FWHM) for the EQE is approximately 100 nm. 

**Figure 7:**
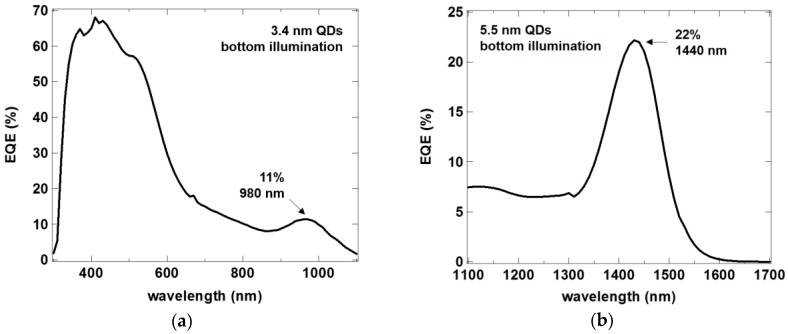
External quantum efficiency of QDPD devices with 3.4 nm (**a**) and 5.5 nm (**b**) quantum dots.

External quantum efficiency of the quantum dot photodetectors extends over a wide range of wavelengths (above 300 nm) and then features a characteristic absorption peak related to the quantum dot size. In the bottom illumination architecture, we obtain 11% EQE at the wavelength of 980 nm for QDs with a diameter of 3.4 nm, with above 60% EQE between 350 nm and 500 nm (Figure 7a). For the larger QDs of 5.5 nm, the peak lies at 1440 nm, with EQE of 22% (Figure 7b). Taking the photodiode dark current as the major noise component, we can obtain specific detectivity (D*) of 3 × 10^11^ Jones.

### 3.3. QDPD on Silicon

**Figure 8:**
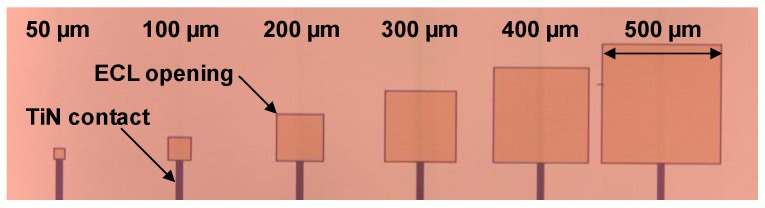
Microscope image of the silicon substrate with different pixel sizes showing TiN bottom contact and inorganic edge cover layer.

After the first optimization of the stack on glass/ITO, the stacks are transferred to 3 × 3 cm^2^ silicon substrates with TiN bottom contact. The contact is structured so that different active areas are available to investigate scaling effects of the dark current. Each substrate has many test vehicles with pixel sizes of 2 × 2 mm^2^ down to 50 × 50 μm^2^. An inorganic edge cover layer (ECL) is used to precisely define the active area and exclude the effects of the fanout structure (Figure 8). 

**Figure 9:**
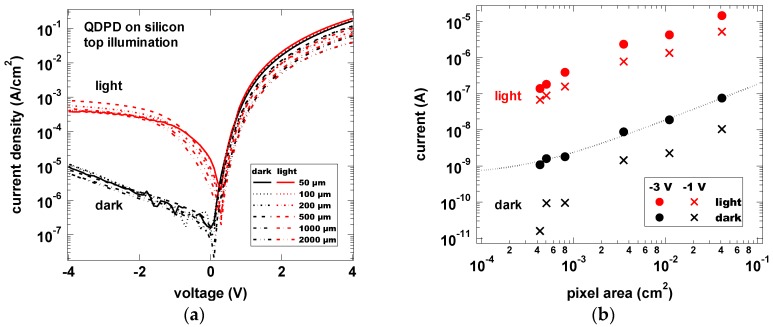
Dark and photocurrent density vs. voltage curves (**a**); and current vs. pixel area curves (**b**) for active areas between 50 × 50 μm^2^ and 2 × 2 mm^2^.

Dark current density on silicon/TiN substrates equals 6 × 10^−3^ mA/cm^2^ at −2 V which shows that the photodetector can be fabricated on the CMOS-compatible bottom contact (Figure 9a). Each pixel size showed also the same current density, indicating linear scaling of the dark current with active area (Figure 9b). Photocurrent was measured with an infrared light emitting diode (5 mW/cm^2^ LED power, 1450 nm center wavelength) in top illumination (through the semi-transparent top contact) and followed the same trend. 

**Figure 10:**
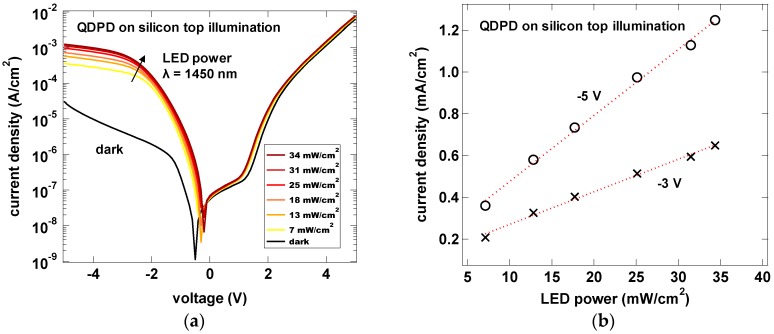
Current density—voltage characteristics (**a**) of a QDPD on Si substrate in dark (black line) and under 1450 nm IR LED with varying power (color lines); and linearity curve of the photocurrent density (**b**).

In top illumination tests with IR LED and increasing irradiance, we could observe a linear increase of the photocurrent (Figure 10). The calculated photo-to-dark current ratio is between 32 and 43 dB in the reverse bias voltage range of −3 to −5 V, respectively. 

**Figure 11:**
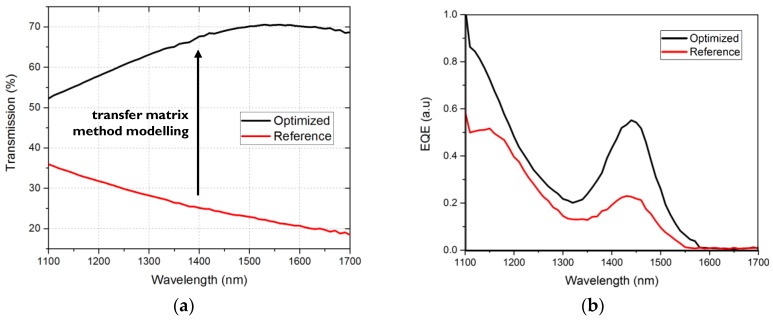
Top illuminated photodetector stack optimized for NIR transparency: Improvement of the top contact transparency (**a**); and experimental EQE verification (**b**).

Indium tin oxide (ITO) bottom contact used in the bottom illuminated test vehicle is not ideal for the top illuminated silicon substrates as it has a limited transparency in the infrared wavelength range (80% at 1100 nm and 55% at 1440 nm). To optimize the semi-transparent top contact for absorption in the NIR range, we used optical interference modelling with transfer matrix method. We obtained transparency of 70% (Figure 11) which was verified experimentally. This shows a significant boost from the standard contact structure used in the visible range. In this way, the EQE of top-illuminated photodetectors on TiN bottom contact can be further improved and reach 25% at the wavelength of 1440 nm, even though the active layer thickness is only 150 nm.

### 3.4. QDPD Additional Characterization

**Figure 12:**
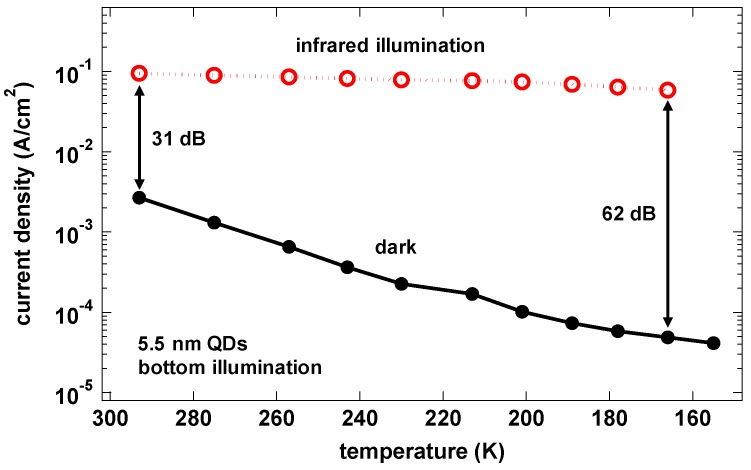
Dark and photocurrent vs. photodetector temperature, indicating an increasing current ratio with lowering the operating temperature.

Even though PbS QDPDs enable detection at room temperature, standard IR camera packaging offers possibility of additional cooling. In the cryostat measurements, we observed that by cooling the detector to 193 K we can further improve the current ratio to over 60 dB from 30 dB at room temperature (Figure 12). Depending on the application, this might be a way to boost the sensitivity of the image sensor. 

**Figure 13:**
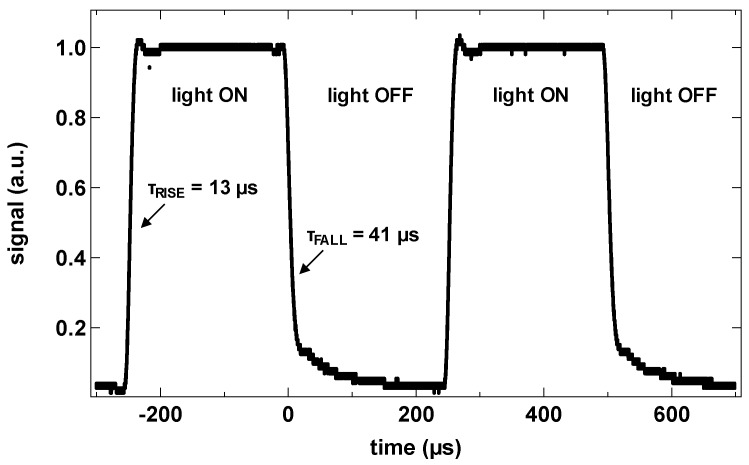
Transient characteristics of a test QDPD device with an active area of 0.041 cm^2^ and under illumination with a switching NIR LED.

Speed of the QDPDs was measured using an oscilloscope for a photodiode with an area of 0.041 cm^2^. While keeping the reverse bias voltage at −2 V, the infrared LED light source (1450 nm center wavelength) was switched on and off. Rise time of 13 μs and fall time of 41 μs were measured (Figure 13). Such performance is sufficient for basic imaging, but a further speed improvement will enable other applications. 

## 4. Discussion

PbS quantum dots are implemented in photodetector stacks targeting detection of near infrared radiation. Dark current density is in the range of 10^−6^ A/cm^2^ at −2 V reverse bias voltage. This value is several orders of magnitude higher than benchmark silicon-based photodetectors (10^−12^ A/cm^2^–10^−10^ A/cm^2^), and within an order of magnitude as compared to InGaAs-based infrared photodetectors operating at similar conditions [2]. Other quantum dot photodetectors show different values of dark current density (10^−8^ A/cm^2^–10^−3^ A/cm^2^), depending on the QD size, ligands, transport layers used in the stack and characterization conditions [16,23,24]. Even though it is difficult to have a direct comparison between published results, the photodetectors presented here have comparable dark current, especially taking into account the stack design for higher cut-off wavelength (thus with a lower energy bandgap). Here, the dark current values are given for the 5.5 nm QDs (1440 nm) and they are expected to be lower for the smaller diameters (e.g., for 940 nm). At the same time, the best organic photodetectors with similar thin-film multilayer stacks feature dark currents comparable to silicon (10^−11^ A/cm^2^ after [11]), indicating that the leakage current might not be limited by the thin-film stack but rather by the narrow-bandgap semiconductor and its interfaces. Current density scales linearly for pixel sizes between 2 × 2 mm^2^ and 50 × 50 μm^2^, indicating no perimeter effects down to this active area. For smaller pixels, active readout is necessary due to the very low current levels. 

External quantum efficiency (EQE) is above 20% at the absorption peak at the wavelength of 1440 nm in a 150-nm thick active layer. Even though this is still significantly lower than EQE of InGaAs photodetectors, it is at the same time unachievable by Si photodetectors. Moreover, we have also demonstrated stacks with other absorption peaks, as they can be quite accurately tuned by the QD diameter. For a 980-nm peak stack, the EQE is above 10% and is currently under optimization, expected to exceed 40–50%. As the QDPD can be integrated on top of the CMOS ROIC, one might imagine not only monochromatic infrared imagers, but also extension of current hyperspectral visible imagers in combination with a standard silicon pinned photodiode. 

As the QDPD stack will be integrated on top of a CMOS ROIC, the pixel stack is optimized for operation in a top illuminated architecture with a TiN bottom contact. We have observed similar electrical performance as in reference devices on glass substrates with ITO contact. Photocurrent and thus efficiency was improved by tuning the thicknesses of all layers in the stack with optical simulations. The transfer matrix method can be used to maximize performance of thin films for the wavelength of interest. We have calculated that the EQE achievable with PbS is about 30% for the wavelength of 1440 nm. 

Even though one of the advantages of the PbS photodetector is operability at room temperature, we have seen that by cooling the device to 193 K, the photo-to-dark current ratio can be increased from 30 dB to 60 dB. This shows the potential of higher sensitivity for specific applications. The photodetector speed is sufficient for imaging, but is still a parameter of concern. Currently, we are investigating possible limiting factors (e.g., trap states, interface defects) and optimizing the charge transfer properties of the stack to reach switching speeds in the range of tens of ns. 

The next step of development is integration of the photodetector stack on the CMOS ROIC test chip. Research activities include lifetime studies (to verify the encapsulation specifications), photolithographic patterning feasibility (to enable side-by-side multicolor arrays) and packaging aspects. The monolithic QDPD integration method will enable low-cost infrared cameras with resolution and pitch limited only by the ROIC design. 

## 5. Conclusions

Stacks using PbS quantum dot absorber layer can be used as efficient near infrared photodetectors even though the total thickness is only in the range of 100 nm. This makes them an interesting candidate for integration on top of CMOS readout circuits, thus enabling monolithic image sensors that have an order-of-magnitude lower cost figure than hybrid devices and are not limited by the resolution and array size of flip-chip hybridization. 

The quantum dot photodetector stack can be fabricated using standard semiconductor processing methods in the fab environment. 8- or 12-inch substrates can be used. In the final camera system, the detailed optical design will strongly depend on the wavelength of interest for the application. For the case of extension of visible CMOS image sensor to NIR, the standard optics found in commercial cameras might be used, while infrared optics will be necessary for a monochrome NIR imager for the higher wavelengths towards 2 μm. 

In summary, the pixel stack demonstrated here shows building blocks for fabrication of a monolithic image sensor for the near infrared wavelength range. The benefits of using QDPD active stack are ease of processing, room-temperature operation, submicron active layer thickness and high EQE in NIR.
